# Anti-echinococcal effect of verapamil involving the regulation of the calcium/calmodulin-dependent protein kinase II response* in vitro* and in a murine infection model

**DOI:** 10.1186/s13071-021-04618-4

**Published:** 2021-02-15

**Authors:** Hai-Jun Gao, Xu-Dong Sun, Yan-Ping Luo, Hua-Sheng Pang, Xing-Ming Ma, Ting Zhang, Tao Jing, Wei Hu, Yu-Juan Shen, Jian-Ping Cao

**Affiliations:** 1 School of Basic Medical Sciences, Lanzhou University, Lanzhou, 730000 Gansu Province People’s Republic of China; 2 National Institute of Parasitic Diseases, Chinese Center for Disease Control and Prevention, WHO Collaborating Center for Tropical Diseases, National Center for International Research on Tropical Diseases, Key Laboratory of Parasite and Vector Biology of the Chinese Ministry of Health, Shanghai, 200025 People’s Republic of China; 3 Ganzr Tibetan Autonomous Prefecture Center for Disease Control and Prevention, Kangding, 626000 Sichuan Province People’s Republic of China; 4 National Health Commission Key Laboratory of Echinococcosis Prevention and Control, Tibet Autonomous Region Center for Disease Control and Prevention, Lhasa, 850000 Tibet Autonomous Region People’s Republic of China

**Keywords:** Verapamil, *Echinococcus*, Calmodulin, Calcium-calmodulin-dependent protein kinases

## Abstract

**Background:**

Echinococcosis, which is caused by the larvae of cestodes of the genus *Echinococcus*, is a parasitic zoonosis that poses a serious threat to the health of humans and animals globally. Albendazole is the drug of choice for the treatment of echinococcosis, but it is difficult to meet clinical goals with this chemotherapy due to its low cure rate and associated side effects after its long-term use. Hence, novel anti-parasitic targets and effective treatment alternatives are urgently needed. A previous study showed that verapamil (Vepm) can suppress the growth of *Echinococcus granulosus* larvae; however, the mechanism of this effect remains unclear. The aim of the present study was to gain insight into the anti-echinococcal effect of Vepm on *Echinococcus* with a particular focus on the regulatory effect of Vepm on calcium/calmodulin-dependent protein kinase II (Ca^2+^/CaM-CaMKII) in infected mice.

**Methods:**

The anti-echinococcal effects of Vepm on *Echinococcus granulosus* protoscoleces (PSC)* in vitro* and *Echinococcus multilocularis* metacestodes in infected mice were assessed. The morphological alterations in *Echinococcus* spp. induced by Vepm were observed by scanning electron microscopy (SEM), and the changes in calcium content in both the parasite and mouse serum and liver were measured by SEM-energy dispersive spectrometry, inductively coupled plasma mass spectrometry and alizarin red staining. Additionally, the changes in the protein and mRNA levels of CaM and CaMKII in infected mice, and in the mRNA levels of *CaMKII* in *E. granulosus* PSC, were evaluated after treatment with Vepm by immunohistochemistry and/or real-time quantitative polymerase chain reaction.

**Results:**

*In vitro*, *E. granulosus* PSC could be killed by Vepm at a concentration of 0.5 μg/ml or higher within 8 days. Under these conditions, the ultrastructure of PSC was damaged, and this damage was accompanied by obvious calcium loss and downregulation of *CaMKII* mRNA expression.* In vivo*, the weight and the calcium content of *E. multilocularis* metacestodes from mice were reduced after treatment with 40 mg/kg Vepm, and an elevation of the calcium content in the sera and livers of infected mice was observed. In addition, downregulation of CaM and CaMKII protein and mRNA expression in the livers of mice infected with *E. multilocularis* metacestodes was found after treatment with Vepm.

**Conclusions:**

Vepm exerted a parasiticidal effect against *Echinococcus* both* in vitro* and* in vivo* through downregulating the expression of Ca^2+^/CaM-CaMKII, which was over-activated by parasitic infection. The results suggest that Ca^2+^/CaM-CaMKII may be a novel drug target, and that Vepm is a potential anti-echinococcal drug for the future control of echinococcosis.
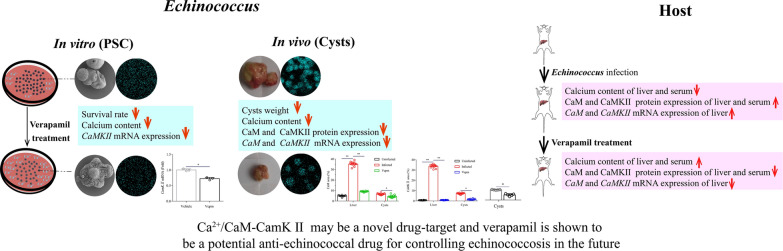

## Background

Echinococcosis is a serious but neglected helminthic zoonosis caused by members of the genus *Echinococcus*, and mainly by *Echinococcus granulosus*, which causes cystic echinococcosis (CE), and *Echinococcus multilocularis*, which causes alveolar echinococcosis (AE) [1]. CE occurs globally, and AE occurs in the northern hemisphere and imposes a heavy disease burden [2]. According to data from central Asia, at least 270 million people are exposed to *Echinococcus*, and the prevalence of the disease in some areas of Tibet in western China ranges from 0.8 to 11.9% [3]. Higher pathogenicity and case fatality rate are notably associated with AE due to its tumor-like growth [3]. The clinical treatment strategies for echinococcosis include surgery and chemotherapy. Albendazole (ABZ), a benzimidazole derivative, is the main chemotherapeutic drug used for the treatment of human echinococcosis [4]; it exerts an anti-parasitic effect by disrupting microtubule polymerization and biochemical processes, such as glucose and energy metabolism, in the parasite [5]. However, ABZ exerts parasitostatic rather than parasiticidal effects [6], has poor gastrointestinal absorption and is associated with severe side effects [7, 8]. Hence, new drugs for the treatment of this parasitosis have been investigated, such as traditional Chinese medicines derived from botanical extracts [9], antineoplastic chemotherapeutics [7, 10], and immunosuppressants [11, 12]. However, only a few of these agents, such as mefloquine and amphotericin B alone or in combination with nitazoxanide, have been used as auxiliary treatments for human echinococcosis [6]. Therefore, investigation of novel anti-*Echinococcus* drugs and drug targets is urgently needed.

Ca^2+^, a pivotal second messenger, controls physiological processes in cells, such as proliferation, differentiation and migration [13]. The complex consisting of Ca^2+^ and calmodulin (CaM) can specifically bind to CaM-dependent protein kinases (CaMKs; including CaMKI, CaMKII, CaMKIII and CaMKIV) to activate the Ca^2+^/CaM-CaMK cascade, which can promote signal transduction in cells [14]. Previous studies in tumors have shown that the Ca^2+^/CaM-CaMK cascade controls tumorigenesis and tumor progression [13, 15, 16]. Furthermore, the Ca^2+^/CaM-CaMK cascade has been suggested to be closely related to the pathogenesis of many hepatic parasites. For example, in *Schistosoma mansoni*, the IQ motif of SmCav1B, which is a voltage-gated calcium channel, can interact with two CaMs, SmCaM1 and SmCaM2, to promote growth [17], and RNA interference silencing of a calcium-regulated protein affects the morphology and vitality of *Schistosoma japonicum* [18]. Two voltage-gated calcium channel β-subunits, CsCa_v_β1 and CsCa_v_β2, boost the parasiticidal effect of praziquantel on *Clonorchis sinensis* [19], and *Fh*CaM dyshomeostasis can clearly block growth and motility in *Fasciola hepatica* [20]. Therefore, Ca^2+^/CaM-CaMKs are potential therapeutic targets in cancers and parasitosis, and calcium channel inhibitors, such as verapamil (Vepm) and praziquantel, have been confirmed to have anti-tumorigenic and anti-parasitic effects [13, 15, 21, 22]. Furthermore, Vepm has been shown to alleviate atrial fibrillation in rats by downregulating the expression of over-activated Cav1.2-CaM-CaMKII [23]. In *E. granulosus* protoscoleces (PSC), the presence of calcareous corpuscles indicate that calcium sources are required for hydatid cyst development [24], and the calcium level in hydatid cysts has been shown to be higher than that in the serum or plasma of the host [25]. Previous studies have confirmed that Vepm suppresses the growth of *E. granulosus* larvae [26, 27]; however, it is unclear whether this compound has similar anti-parasitic effects on *E. multilocularis*, which has a tumor-like larval stage, and is more harmful to humans. The mechanism of action of Vepm against *Echinococcus* has never been investigated, and it is unclear whether the inhibitory effects of Vepm on *E. granulosus* are mediated by the regulation of Ca^2+^/CaM-CaMKII. Hence, this study aimed to investigate the efficacy and mechanism of action of Vepm against *Echinococcus*.

## Methods

### Biochemical reagents

Vepm (V111249), ABZ (A131023), albendazole sulfoxide (ABZ-SO; 35395) and pentobarbital sodium were purchased from Sigma (MI) and Aladdin (Shanghai, China). Antibodies and polymerase chain reaction (PCR) primers were purchased from Abcam (Cambridge, MA) and Beijing Genomics Institute (Beijing). Enzyme-linked immunosorbent assay (ELISA) kits for CaM and CaMKII were purchased from CUSABIO (Wuhan, China). All culture reagents were purchased from Gibco (Wisent, Canada). Extraction kits for total RNA and PCR kits were purchased from Takara (Tokyo).

### Separation and culture of* E. granulosus* PSC* in vitro*

*E. granulosus* PSC were obtained from the livers of naturally infected sheep from the slaughterhouse of Xining City, Qinghai Province, China. The PSC were rinsed with phosphate buffered saline (PBS), resuspended in Dulbecco’s modified Eagle’s medium containing 1% penicillin-streptomycin, and then incubated in 24-well culture plates (100 PSC/well) at 37 ℃, 5% CO_2_. Morphological alterations in the PSC were observed under an inverted microscopy (BX43; Olympus, Japan), and the survival rate of PSC was recorded daily.

### Mouse infection with* E. multilocularis*

Kunming mice 6–8 weeks of age were purchased from the Laboratory Animal Center of Lanzhou University and housed in a high-efficiency particulate air-filtered and temperature-controlled environment under a light/dark cycle at 22-25 ℃. The mice were fed a rodent diet (Beijing Keao; Beijing) *ad libitum* under specific-pathogen-free laboratory conditions. *E. multilocularis* PSC were aseptically isolated from anaesthetized *E. multilocularis*-infected gerbils in our laboratory as described previously [28] to establish a murine infection model by* in situ* surgical intrahepatic implantation. Healthy mice were infected with *E. multilocularis* PSC (1500 PSC/mouse) (*n* = 15) under specific-pathogen-free laboratory conditions, or were subjected to a sham procedure and used as the uninfected group (*n* = 5); both groups were housed under the same conditions.

### Drug treatment* in vitro*

The experimental groups were divided into (i) the vehicle group, which was treated with 0.1% dimethylsulfoxide (DMSO) (*n* = 3); (ii) the ABZ-SO group, which was treated with 40 and 20 μg/ml ABZ-SO dissolved in 0.1% DMSO (*n* = 3); and (iii) the Vepm groups, which were treated with 100, 80, 40, 20, 10, 5, 2, 1 or 0.5 μg/ml Vepm (*n* = 3). After drug treatment, *E. granulosus* PSC were stained with 0.4% trypan blue for 10 min to observe morphological changes. In addition, *E. granulosus* PSC were fixed with 4% glutaraldehyde, rinsed with PBS (1×), and stained with 2% osmium tetroxide for 2 h and 1% uranyl acetate for 30 min. Subsequently, these specimens were dehydrated in increasing concentrations of ethanol, air-dried and coated with gold as described previously [28]. Finally, the microstructure of the PSC was observed by scanning electron microscopy (SEM) (JSM-5600LV; JEOL, Japan).

### Drug treatment* in vivo*

Three months after infection with *E. multilocularis* PSC, the infected mice were divided into the following groups for oral drug administration: (i) infected mice treated daily with honey/PBS (1:1 v/v) (*n* = 5); (ii) infected mice treated daily with 40 mg/kg ABZ in honey/PBS (1:1 v/v) (*n* = 5); (iii) infected mice treated daily with 40 mg/kg Vepm in honey/PBS (1:1 v/v) (*n* = 5). (iv) The uninfected mice were treated daily with honey/PBS (1:1 v/v) (*n* = 5). After 4 months of treatment, *E. multilocularis* cysts, sera and livers were harvested from the mice to measure the calcium content and CaM and CaMKII protein and mRNA expression.

### Calcium content analysis by inductively coupled plasma mass spectrometry, SEM-energy dispersive spectrometry and alizarin red staining

i. For inductively coupled plasma mass spectrometry (ICP-MS) analysis, equal amounts of tissue or serum/culture supernatant samples were prepared for calcium analysis. Tissue digestion was performed with a microwave digestion system using UltraClave (Milestone; Sorisole, Italy), and sample analysis was performed as described previously [29].
ii. For SEM-energy dispersive X-ray spectroscopy (SEM-EDS) analysis, changes in the calcium content in *E. granulosus* PSC and in the germinal layer cells of *E. multilocularis* metacestodes were observed after treatment with Vepm by a LEO Gemini field emission gun scanning electron microscope (JEOL, Japan) as described previously [30].
iii. For alizarin red staining, *E. multilocularis* metacestodes and mouse livers were fixed with 4% paraformaldehyde for 2 weeks, and then stained with alizarin red to detect calcification, as described previously [31]. The percentage of positively stained calcium deposits in the images was calculated by using ImageJ software (National Institutes of Health, Bethesda, MD).

### Analysis of CaM and CaMKII protein expression analysis by immunohistochemistry-paraffin and ELISA

i. For immunohistochemistry-paraffin (IHC-P), 4-μm-thick sections of *E. multilocularis* metacestodes and mouse livers were processed to evaluate the expression of CaM or CaMKII, as described previously [32, 33]; immunostaining was performed using rabbit anti-CaM/CaMKII antibodies (Bioss; Beijing) at 1:300/1:200 dilution and secondary antibodies (goat anti-rabbit immunoglobulin G; Bioss) at 1:800 dilution. Finally, the slides were imaged under a fluorescence microscopy (Olympus, Japan). Semiquantitative analysis was performed by using ImageJ software.
ii. For ELISA, PBS (pH = 7.2) containing phenylmethylsulfonyl fluoride (10 mmol/l) was added to serum and tissue samples, which were then rapidly homogenized and centrifuged at 1000×* g* for 10 min to measure the protein concentrations of CaM and CaMKII according to the ELISA kit protocols.

### Analysis of CaM and CaMKII mRNA expression by real-time quantitative PCR

The expression of *CaM* or/and *CaMKII* mRNA in *E. granulosus* PSC, mouse livers and *E. multilocularis* cysts was measured by real-time quantitative PCR (RT-qPCR), and *β-actin* mRNA was used as the internal standard. Nucleic acid was isolated from various tissues by using TRIzol reagent (Invitrogen, San Diego, CA), and reverse-transcribed into cDNA; amplification of cDNA was performed by RT-qPCR following the Takara kit protocol (no. RR036A). The following primers were used: *Eg*-*CaMKII/Em*-*CaMKII* (forward, 5′-TCGTTGTTCAAGTCGGTTCG-3′; reverse, 5′-GGTGCTGAGAGACCCACTAG-3′), *Eg*-*β*-*actin/Em*-*β*-*actin* (forward, 5′-AGACATCAGGGAGTGATGGTT-3′; reverse, 5′-GAGGACTGGATGCTCCTCAGG-3′); mouse *CaMKII* (forward, 5′-GGCCTGGACTTTCATCGATTCTA-3′; reverse, 5′-CATCAGGTGGATGTGAGGGTTC-3′), mouse *CaM* (forward, 5′-AAGCCGAGCTGCAGGATATGA-3′; reverse, 5′-CAGTTCTGCCGCACTGATGTAA-3′), and mouse *β-actin* (forward, 5′-TTGTTACCAACTGGGACG-3′; reverse, 5′-GGCATAGAGGTCTTTACGG-3′).

### Statistical analysis

The data are presented as the mean ± SD. Statistical differences between the groups were assessed by* t*-test and paired comparisons. Statistical analysis was performed by SPSS version 22.0 (IBM, Chicago, IL) and GraphPad Prism version 7.0 (GraphPad Software, San Diego, CA). *P* < 0.05 indicates significant differences.

## Results

### Effect of Vepm on* E. granulosus* PSC* in vitro*

**Fig. 1a, b:**
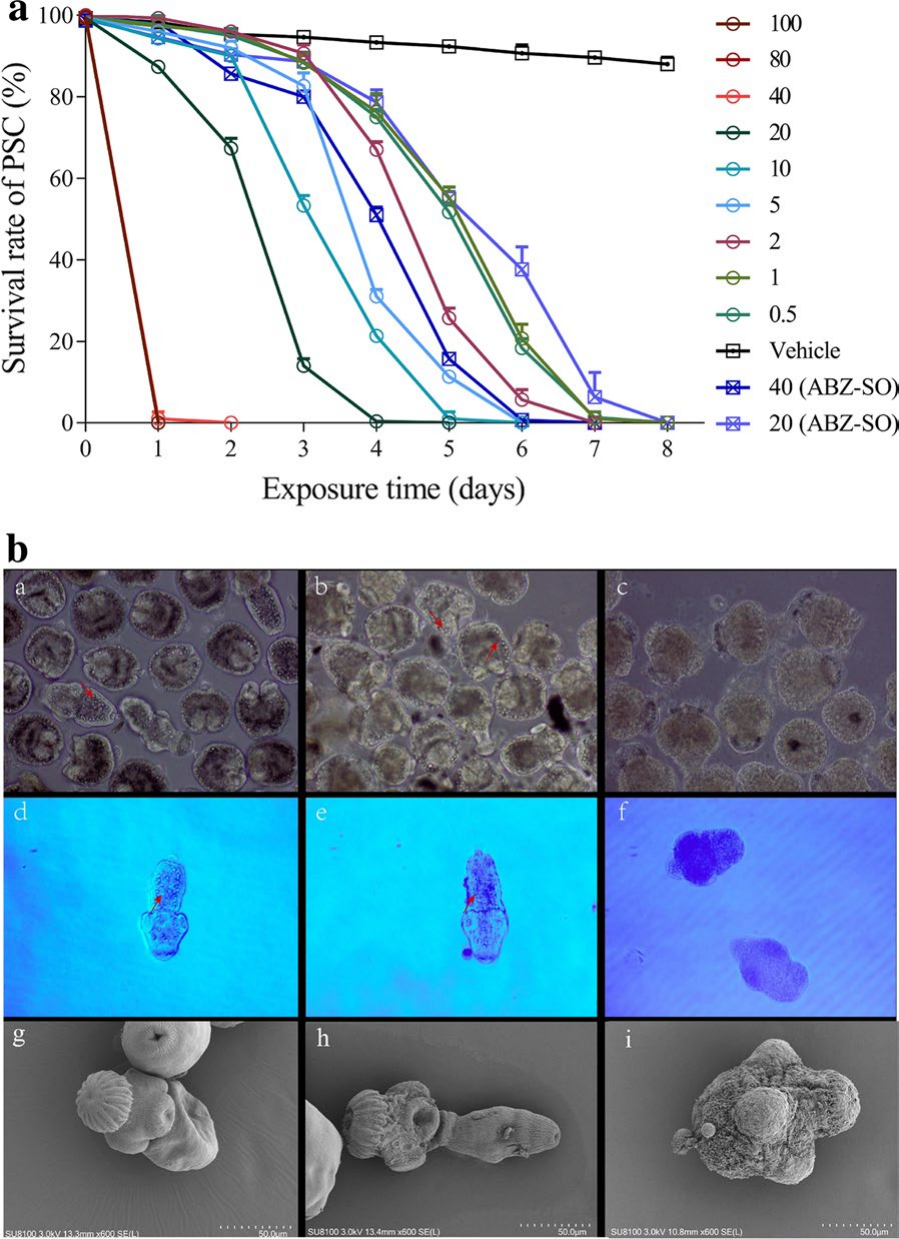
The effect of verapamil (Vepm) against *Echinococcus granulosus* protoscoleces (*PSC*)* in vitro*.** a** *E. granulosus* PSC were exposed to Vepm at a concentration of 0.5–100 μg/ml, albendazole sulfoxide (*ABZ-SO*) at a concentration of 40 or 20 μg/ml, or 0.1% dimethylsulfoxide (the vehicle group) for 8 days.** b** The morphological changes and ultrastructural alterations in PSC exposed to various drugs for 4 days were detected by light microscopy and scanning electron microscopy (SEM).* a*,* d*,* g* Typical morphology and structural integrity of PSC from the vehicle group.* b*,* e*,* h* Mild morphological and structural alterations in the ABZ-SO (20 μg/ml) group.* c*,* f*,* j* Substantial morphological and ultrastructural destruction of PSC exposed to Vepm (20 μg/ml), including disappearance of calcareous corpuscles and hooks, shedding of the tegument and the presence of numerous blebs.* a*–*c* Light microscopy without any staining (×200 magnification);* d*–*f* light microscopy with trypan blue staining (×200 magnification);* g*–*i* SEM (×600 magnification). *Red arrowhead* indicates an intact calcareous corpuscle

The survival rate of *E. granulosus* PSC after treatment with various concentrations of Vepm (0.5, 1, 2, 5, 10, 20, 40, 80 or 100 μg/ml) within 8 days is shown in Fig. 1a. The mortality of PSC upon exposure to 0.5–40 μg/ml Vepm was time dependent; all PSC were killed within 2 days by 40 μg/ml Vepm or within 4 days by 20 μg/ml Vepm. However, ABZ-SO had killed only 13% of PSC on day 2 when administrated at a concentration of 40 μg/ml and 21% of PSC on day 4 when administrated at a concentration of 20 μg/ml; 4% of PSC in the vehicle group were dead on day 2, and 8% of PSC were dead on day 4.

Under light microscopy, comparison with the natural morphology of PSC in the vehicle group indicated mild morphological alterations of PSC in the ABZ-SO (20 μg/ml) group and substantial morphological alterations (i.e., disappearance of calcareous corpuscles) of PSC after exposure to 20 μg/ml Vepm for 4 days (Fig. 1b,* a*–*c*). Additionally, PSC in the Vepm (20 μg/ml) group were stained with trypan blue on day 4, unlike the viable PSC in the ABZ-SO (20 μg/ml) and vehicle groups (Fig. 1b,* d*–*f*). SEM showed that the ultrastructural changes that occurred in PSC exposed to Vepm (20 μg/ml) for 4 days were shedding of the tegument, disappearance of hooks and the presence of numerous blebs; however, PSC in the vehicle and ABZ-SO (20 μg/ml) groups remained intact (Fig. 1b,* g*-*i*).

### Changes in the calcium content in* E. granulosus* PSC exposed to Vepm* in vitro*

**Fig. 2a–c:**
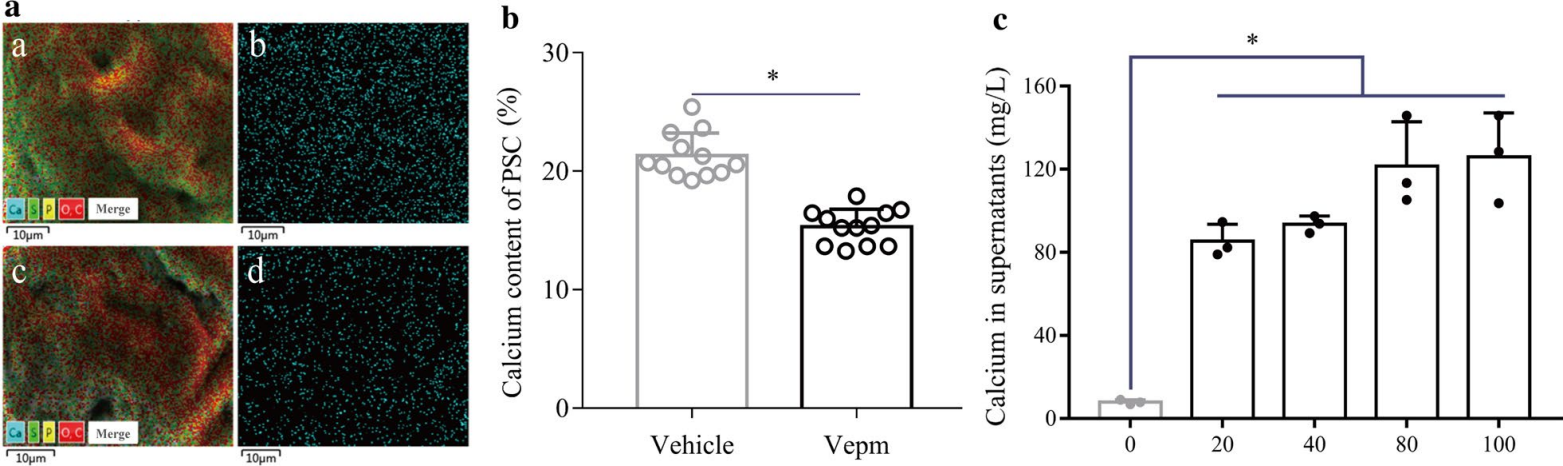
Changes in the calcium content in *E. granulosus* PSC after treatment with 20 µg/ml Vepm for 4 days* in vitro*.** a** Calcium distribution in *E. granulosus* PSC, as assayed by SEM–energy dispersive X-ray spectrometry (SEM-EDS). Merged image of multi-element (*a*,* c*) and calcium (*b*,* d*) distributions in PSC in the vehicle (*a*,* b*) and the Vepm groups (*c*,* d*).** b** Percentage of calcium content in *E. granulosus* PSC, as measured by SEM-EDS.** c** Calcium content in the culture supernatants of *E. granulosus* PSC exposed to Vepm (0, 20, 40, 80, 100 µg/ml) as measured by inductively coupled plasma mass spectrometry. For other abbreviations, see Fig. 1

The calcium distribution in *E. granulosus* PSC treated with Vepm (20 μg/ml) for 4 days was heterogeneous and sparse compared with that in the vehicle group, as determined by SEM-EDS (Fig. 2a). Semiquantitative analysis showed that the calcium level in PSC decreased after treatment with Vepm (Fig. 2b). In addition, a clear dose-dependent increase in the calcium content in the culture supernatant of the Vepm group compared with the vehicle group was observed (Fig. 2c).

### Effect of Vepm on *E. multilocularis* metacestodes* in vivo*

*E. multilocularis*-infected mice were treated with Vepm (40 mg/kg) or ABZ (40 mg/kg) for 4 months* in vivo*. The wet weights of *E. multilocularis* cysts isolated from the Vepm (0.98 ± 0.33 g) and ABZ groups (1.04 ± 0.14 g) were significantly lower than those from the infected group (5.90 ± 0.75 g) (*P* = 0.000; Table 1).

**Table 1:** Changes in the wet weight of *Echinococcus multilocularis* metacestodes in mice after treatment with verapamil (*Vepm*) for 4 months

Group	No. of mice	Dose	Cyst weight (g) (mean ± SD)
Infected	5	NA	5.90 ± 0.75
ABZ	5	40 mg/kg per day	1.04 ± 0.14^*^
Vepm	5	40 mg/kg per day	0.98 ± 0.33^*^

*ABZ* Albendazole, *NA* not available

* *P <* 0.05 (statistical analysis was performed by comparison with the infected group)

### Changes in the calcium concentration in *E. multilocularis*-infected mice after Vepm treatment

**Fig. 3a, b:**
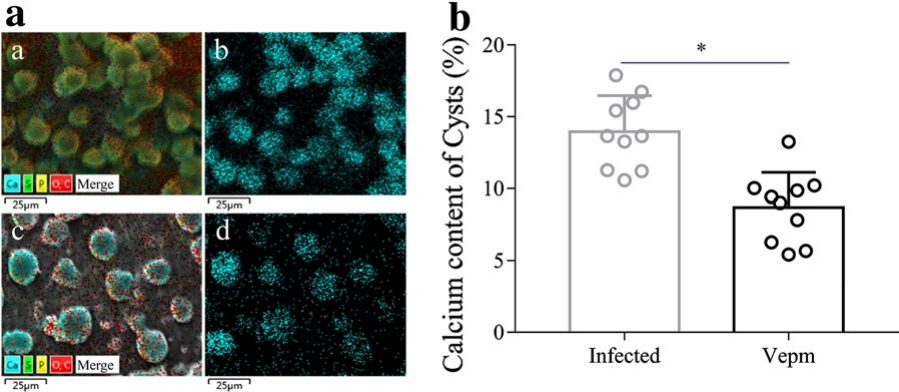
Changes in the calcium content of *E. multilocularis*-infected mice after treatment with 40 mg/kg Vepm for 4 months, as measured by SEM-EDS. **a** Calcium distribution in the germinal layer of *E. multilocularis* cysts. Merged image of multi-element (*a*,* c*) and calcium (*b*,* d*) distributions in the germinal layer of the infected (*a*,* b*) and Vepm groups (*c*,* d*). **b** Percentage of calcium content in the germinal layer of *E. multilocularis* metacestodes in mice after treatment with Vepm. For abbreviations, see Figs. 1 and 2

**Fig. 4a, b:**
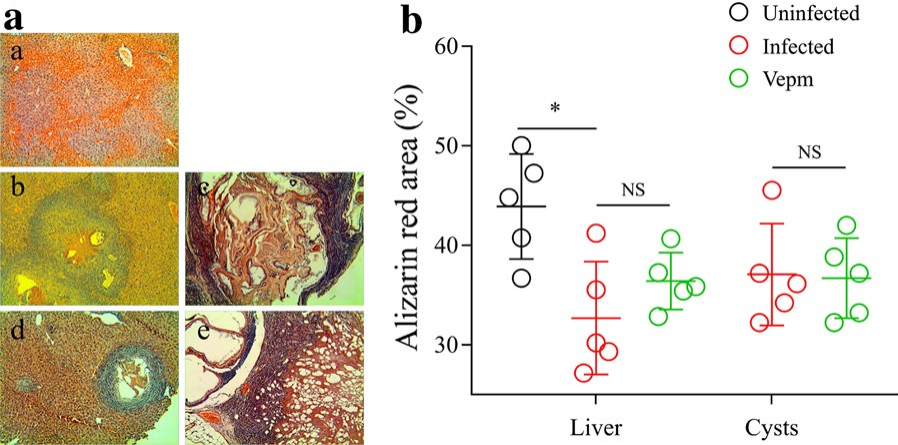
Changes in the calcium content in the liver and *E. multilocularis* cysts in mice after treatment with 40 mg/kg Vepm for 4 months, as measured by alizarin red staining. **a** Calcium distribution in the liver (*a*,* b*,* d*) and *E. multilocularis* cysts (*c*,* e*) from uninfected (*a*), infected (*b*,* c*) and Vepm-treated (*d*,* e*) mice (×200 magnification). **b** Semiquantitative analysis of calcium content.* NS* Not significant

The calcium concentration in the sera of *E. multilocularis*-infected mice was 4.92 ± 0.77 mg/l, which represented a twofold decrease compared with that in the uninfected group (9.89 ± 1.92 mg/l); treatment with Vepm for 4 months induced a recovery of the calcium concentration to 6.39 ± 0.79 mg/l (Table 2). Similarly, the level of calcium in the livers of mice decreased, from 296.72 ± 9.43 mg/l to 172.72 ± 16.63 mg/l, due to *E. multilocularis* metacestode infection. Interestingly, the calcium content in the livers increased to 226.78 ± 43.93 mg/l after treatment with Vepm (*P* = 0.009). The ICP-MS assay showed that the calcium level in *E. multilocularis* cysts isolated from the infected group was 3182.28 ± 190.77 mg/l; the level decreased to 3013.98 ± 115.80 mg/l after treatment with Vepm for 4 months (*P* = 0.13). Additionally, changes in the calcium level in *E. multilocularis* cysts were evaluated by SEM-EDS (Fig. 3a), which showed that the percentage of calcium by weight in *E. multilocularis* cysts rapidly decreased from 14.28% to 8.66% after treatment with Vepm; these differences were statistically significant (*P* = 0.000) (Fig. 3b). The results of alizarin red staining showed that calcium deposition around the portal area of the liver in infected mice was significantly increased compared with that in the uninfected group (Fig. 4). The decrease in calcium content in the infected livers was not reversed by treatment with Vepm. The calcium content in *E. multilocularis* cysts was not obviously reduced (*P* > 0.05); however, a reduction in the number of PSC was observed.

**Table 2:** Inductively coupled plasma mass spectrometry analysis of the calcium content in the sera, livers and cysts of *E. multilocularis*-infected mice after treatment with Vepm for 4 months

Sample	Calcium concentration (mg/l)		
	Uninfected group	Infected group^a^	Vepm group^b^
Serum	9.89 ± 1.92	4.92 ± 0.77^*^	6.39 ± 0.79
Liver	296.72 ± 9.43	172.72 ± 16.63^*^	226.78 ± 43.93^*^
Cyst	NA	3182.28 ± 190.77	3013.98 ± 115.80

^*^P < 0.05

^a^Infected group vs. the uninfected group

^b^Vepm group vs. the infected group

### Analysis of CaM and CaMKII protein levels in *E. multilocularis*-infected mice treated with Vepm

**Fig. 5:**
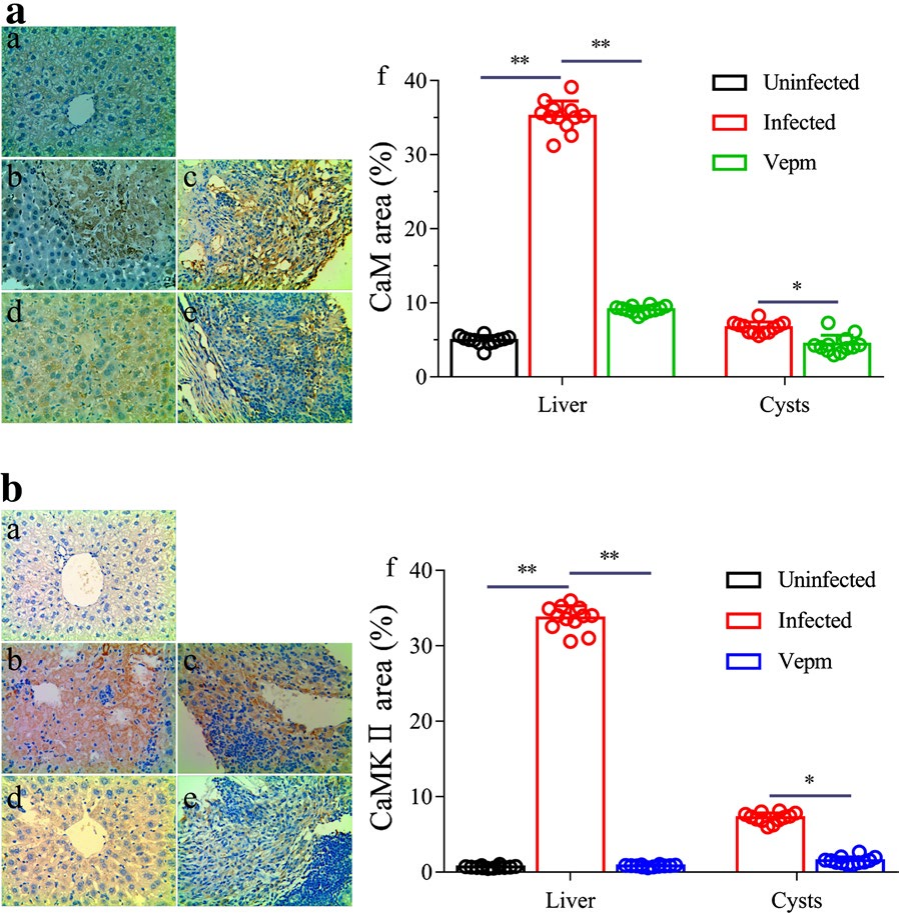
Analysis of calmodulin (*CaM*) and Ca^2+^/calmodulin-dependent protein kinase II (*CaMKII*) protein expression in the liver and *E. multilocularis* cysts after treatment with Vepm, as determined by immunohistochemistry–paraffin. The protein expression of CaM (**a**) and CaMKII (**b**) in the livers (*a*,* b*,* d*) and *E. multilocularis* cysts (*c*,* e*) of uninfected (*a*), infected (*b*,* c*) and Vepm-treated (*d*,* e*) mice (×400 magnification).* f* Semiquantitative analysis of CaM and CaMKII protein expression

IHC-P staining revealed high expression of CaM in the livers of infected mice compared with those of uninfected mice (Fig. 5a); however, pathological progression was inhibited by Vepm, and the differences were statistically significant (*P* < 0.05). Furthermore, ELISA showed that the CaM protein concentration in mouse serum and liver increased significantly from 13.81 ± 1.65 to 22.25 ± 5.55 μg/ml and from 3.42 ± 0.27 to 6.06 ± 1.83 μg/ml, respectively, after *E. multilocularis* infection; however, after treatment with Vepm, CaM protein expression was clearly inhibited in the serum (8.13 ± 1.26 μg/ml) and liver (1.60 ± 0.68 μg/ml) but not in the cysts (from 2.36 ± 0.87 to 1.68 ± 0.10 μg/ml) (Table 3).

**Table 3:** Analysis of calmodulin (*CaM*) and Ca^2+^/calmodulin-dependent protein kinase II (*CaMKII*) protein levels in *E. multilocularis*-infected mice after oral administration of Vepm for 4 months by enzyme-linked immunosorbent assay

Index	Sample	Uninfected group	Infected group^a^	Vepm group^b^
CaM (μg/ml)	Serum	13.81 ± 1.65	22.25 ± 5.55^*^	8.13 ± 1.26^*^
	Liver	3.42 ± 0.27	6.06 ± 1.83^*^	1.60 ± 0.68^*^
	Cysts	NA	2.36 ± 0.87	1.68 ± 0.10
CaMKII (ng/ml)	Serum	5.88 ± 0.99	22.87 ± 4.23^*^	5.13 ± 1.74^*^
	Liver	2.00 ± 0.61	4.25 ± 1.84^*^	1.97 ± 0.56^*^
	Cysts	NA	2.65 ± 1.24	1.79 ± 0.36

^*^P < 0.05

^a^Infected group vs. the uninfected group

^b^Vepm group vs. the infected group

Similar to that of CaM, the IHC-P assay indicated that the overexpression of CaMKII in the livers and cysts in *E. multilocularis*-infected mice was significantly suppressed by Vepm treatment (Fig. 5b). Additionally, an increase in CaMKII content in mouse serum (22.87 ± 4.23 ng/ml) and liver (4.25 ± 1.84 ng/ml) was observed after *E. multilocularis* infection; the CaMKII content decreased to 5.13 ± 1.74 ng/ml and 1.97 ± 0.56 ng/ml, respectively, after Vepm treatment. However, only a mild decrease in CaMKII content in the cysts (from 2.65 ± 1.24 to 1.79 ± 0.36 ng/ml) was observed after Vepm treatment (*P* > 0.05; Table 3).

### Analysis of CaM and CaMKII mRNA expression in the parasite and in *E. multilocularis*-infected mice after treatment with Vepm

**Fig. 6a–c:**
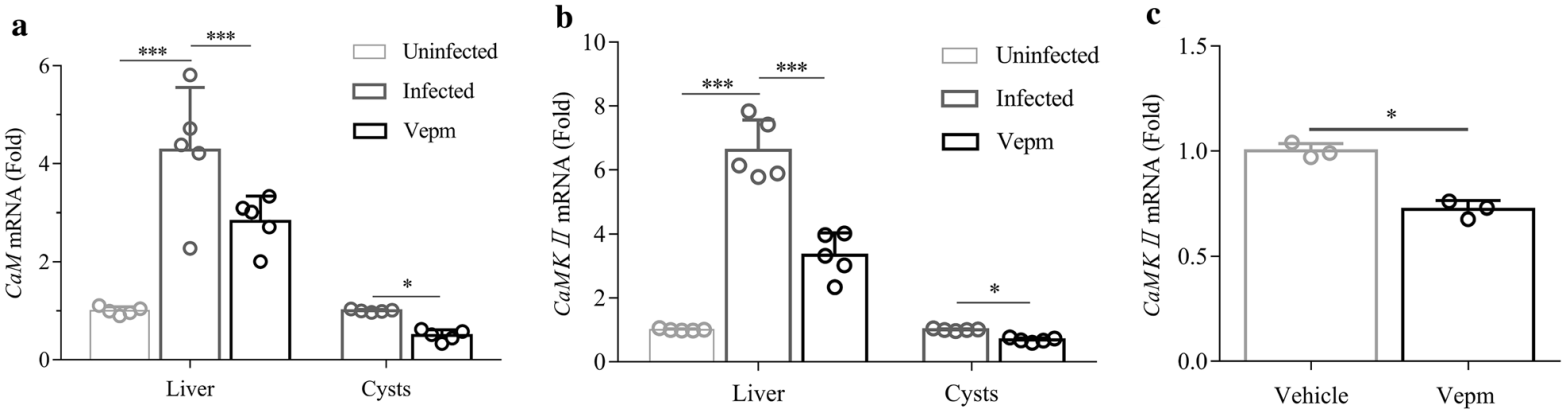
Expression of *CaM* and *CaMKII* mRNA in the livers and *E. multilocularis* cysts of mice after treatment with 40 mg/kg Vepm for 4 months and in *E. granulosus* PSC after exposure to Vepm (20 μg/ml) for 4 days, as measured by real-time quantitative polymerase chain reaction.** a** *CaM* mRNA expression,** b** *CaMKII* mRNA expression,** c** *CaMKII* mRNA expression in *E. granulosus* PSC. For abbreviations, see Figs. 1 and 5

The changes in Ca^2+^/CaM-CaMKII expression in parasite-infected mice after treatment with Vepm were evaluated based on the changes in *CaM* and/or *CaMKII* mRNA expression in the mouse liver and in *Echinococcus*, as measured by using RT-qPCR. *CaM* and *CaMKII* mRNA expression in the mouse liver increased fourfold and sixfold, respectively, after infection with *Echinococcus* (Fig. 6a, b). Overexpression of *CaM* and *CaMKII* mRNA in the liver was significantly suppressed by Vepm (*P* = 0.000). After treatment with Vepm, *CaMKII* mRNA expression was clearly downregulated, while *CaM* mRNA expression was mildly reduced (*P* > 0.05). Furthermore, *CaMKII* mRNA expression in *E. granulosus* PSC exposed to Vepm* in vitro* was clearly downregulated (Fig. 6c).

## Discussion

*Echinococcus granulosus and Echinococcus multilocularis* metacestodes tend to parasitize the liver, and *E. multilocularis* metacestodes, which exhibit tumor-like growth, become more noticeable and lead to death if untreated [34]. While its intestinal absorption is poor and it is associated with severe side effects [7], ABZ, which exerts a parasitostatic rather than a parasiticidal effect, has long been used to treat human echinococcosis. Hence, new anti-echinococcal targets and therapeutic options should be urgently explored.

The Ca^2+^/CaM-CaMK pathway is a potential therapeutic target for cancers, and is closely monitored in many cases of parasitosis. However, as it is unclear how Ca^2+^/CaM-CaMKII regulates the growth and development of *Echinococcus* spp., this needs to be explored to identify potential drugs for the treatment of echinococcosis at early stages of the disease.

The results of the present study indicated that the anti-echinococcal effect of Vepm on *E. granulosus* PSC was time- and dose-dependent, similar to the inhibitory effects of this drug on tumor proliferation. Calcareous corpuscles in PSC can persistently provide abundant calcium sources to promote the development of PSC into cysts [24]. In the present study, rapid disappearance of calcareous corpuscles and a decrease in the calcium content in *E. granulosus* PSC were induced by exposure to Vepm, indicating that Vepm kills *E. granulosus* PSC by promoting calcium loss.

AE is often called “parasitic cancer” due to the distinctive tumor-like growth (metacestode) of *E. multilocularis* larvae; in AE, approximately 70% of the metacestodes are found in the right lobe of the patient’s liver. The germinal layer cells in *E. multilocularis* metacestodes have high regenerative capacity and can develop into new multicellular structures, such as PSC, which can further develop into new metacestodes. Therefore, *E. multilocularis* metacestodes can infiltrate the whole liver of the host [35]. In this study, an infection model was established by *in situ* surgical intrahepatic implantation to reproduce the natural onset and development of AE in mice as closely as possible. After *E. multilocularis*-infected mice were treated with 40 mg/kg Vepm for 4 months, the weight of *E. multilocularis* cysts was significantly decreased, suggesting that the growth of the metacestodes was suppressed. This finding is similar to those reported by Cao on the effect of Vepm in mice with CE [26]. Infection with *E. multilocularis* metacestodes was reported to reduce serum calcium levels in both mice and humans [25, 36]. Interestingly, our results revealed that the reduction in the calcium content in *E. multilocularis*-infected mice was significantly reversed after administration of Vepm, and an apparent mild increase was observed in the liver but not in the serum or cysts. The calcium content in *E. multilocularis* metacestodes was substantially higher than those in the host liver and serum, indicating that the development of *E. multilocularis* PSC into metacestodes requires continuous absorption of calcium from the host. Furthermore, the results of the SEM-EDS assay showed that treatment with Vepm clearly reduced the calcium content and the number of germinal layer cells. Thus, we speculate that the proliferation of germinal layer cells is regulated by calcium supply in infected mice and can be disrupted by the calcium channel inhibitor Vepm. However, the detailed mechanism by which Vepm suppresses the growth of germinal layer cells by regulating the Ca^2+^/CaM-CaMK cascade requires additional investigation. Furthermore, the results of alizarin red staining and ICP-MS suggested that *E. multilocularis* metacestodes cause calcium translocation from mice into the parasite, and that a calcium channel inhibitor (Vepm) reverses calcium loss in *E. multilocularis*-infected mice. In addition, abundant lymphocytes surrounded the *E. multilocularis* cysts in the liver after treatment with Vepm, which may have been associated with an increase in the calcium content in the livers of *E. multilocularis*-infected mice after treatment with Vepm, probably because a sufficient level of calcium can promote the proliferation and polarization of T cells [37]. However, the abnormal changes in lymphocyte development caused by calcium loss in *Echinococcus*-infected hosts requires further investigation.

Our results indicated that an increase in the expression of CaM and CaMKII proteins in mouse serum and liver was caused by *E. multilocularis* metacestode infection; however, overexpression of the CaM and CaMKII proteins was significantly suppressed by Vepm. Furthermore, the expression of *CaM* and *CaMKII* mRNA in the mouse liver and in *E. multilocularis* metacestodes was downregulated after treatment with Vepm. Our results partially support the recent findings of Nawaratna, who showed that Ca^2+^/CaM-CaMKII is a putative therapeutic target for helminth parasite infection that can provide biochemical and pharmacological information for exploring novel compounds in the future [38]. Ca^2+^/CaM-CaMKII controls the completion of the life cycle in *Echinococcus*, and the calcium channel blocker Vepm has strong inhibitory effects against *Echinococcus*, including their germinal layer cells, PSC and metacestodes. Therefore, subsequent studies should evaluate the molecular and immune mechanisms of the effects of Vepm on the over-activation of the Ca^2+^/CaM-CaMKII pathway in *Echinococcus*-infected hosts. Furthermore, novel anti-echinococcal drug targets and effective treatment strategies associated with Ca^2+^/CaM-CaMKII should be carefully investigated.

## Conclusions

The results of our study suggested that Vepm has parasiticidal effects on *E. granulosus* PSC* in vitro* by promoting calcium loss, and on *E. multilocularis* metacestodes* in vivo* by inhibiting calcium translocation from mice to parasites. Furthermore, Ca^2+^/CaM-CaMKII in mice is over-activated by *Echinococcus* infection, and this effect is alleviated by oral administration of Vepm. Thus, our findings provide new information by identifying Ca^2+^/CaM-CaMKII as the target of the anti-*Echinococcus* effects of Vepm. Investigation of the functions of Ca^2+^/CaM-CaMKII in the growth and development of *Echinococcus* and the chronic toxicity of Vepm is currently in progress in our laboratory.

## Data Availability

The datasets supporting the conclusions of this article are included within the article.

## Abbreviations

ABZ: Albendazole
ABZ-SO: Albendazole sulfoxide
AE: Alveolar echinococcosis
CaM: Calmodulin
CaMKII: Ca^2+^/calmodulin-dependent protein kinase II
CE: Cystic echinococcosis
DMSO: Dimethylsulfoxide
ELISA: Enzyme-linked immunosorbent assay
ICP-MS: Inductively coupled plasma mass spectrometry
IHC-P: Immunohistochemistry-paraffin
PBS: Phosphate buffered saline
PSC: Protoscoleces
RT-qPCR: Real-time quantitative polymerase chain reaction
SEM-EDS: Scanning electron microscopy–energy dispersive X-ray spectrometry
Vepm: Verapamil
